# Transcriptome organization of white blood cells through gene co-expression network analysis in a large RNA-seq dataset

**DOI:** 10.3389/fimmu.2024.1350111

**Published:** 2024-04-02

**Authors:** Paola Forabosco, Mauro Pala, Francesca Crobu, Maria Antonietta Diana, Mara Marongiu, Roberto Cusano, Andrea Angius, Maristella Steri, Valeria Orrù, David Schlessinger, Edoardo Fiorillo, Marcella Devoto, Francesco Cucca

**Affiliations:** ^1^ Istituto di Ricerca Genetica e Biomedica (IRGB), Consiglio Nazionale delle Ricerche (CNR), Cagliari, Italy; ^2^ CRS4-Next Generation Sequencing (NGS) Core, Parco POLARIS, Cagliari, Italy; ^3^ Laboratory of Genetics and Genomics, National Institute on Aging, National Institutes of Health (NIH), Baltimore, MA, United States; ^4^ Dipartimento di Medicina Traslazionale e di Precisione, Università Sapienza, Roma, Italy; ^5^ Dipartimento di Scienze Biomediche, Università degli Studi di Sassari, Sassari, Italy

**Keywords:** immune system, network analysis, WGCNA, lncRNA, RNA-seq, white blood cells

## Abstract

Gene co-expression network analysis enables identification of biologically meaningful clusters of co-regulated genes (modules) in an unsupervised manner. We present here the largest study conducted thus far of co-expression networks in white blood cells (WBC) based on RNA-seq data from 624 individuals. We identify 41 modules, 13 of them related to specific immune-related functions and cell types (e.g. neutrophils, B and T cells, NK cells, and plasmacytoid dendritic cells); we highlight biologically relevant lncRNAs for each annotated module of co-expressed genes. We further characterize with unprecedented resolution the modules in T cell sub-types, through the availability of 95 immune phenotypes obtained by flow cytometry in the same individuals. This study provides novel insights into the transcriptional architecture of human leukocytes, showing how network analysis can advance our understanding of coding and non-coding gene interactions in immune system cells.

## Introduction

Systems biology approaches are used to elucidate patterns of transcriptome organization by identifying how genes function jointly to form subsystems (1). Gene co-expression networks are commonly used and powerful analyses to interpret transcriptome data, based on the assumption that genes which are co-expressed belong to the same subsystem and have a higher probability of having related functions, because genes whose products work together must logically be expressed together (2). It is therefore often assumed that observed co-expression results from co-regulation (3), i.e. the coordinated transcription of genes in a program of regulatory mechanisms within a cell, providing the foundation for understanding genome function.

Co-expression network analysis thus identifies gene sets, or modules, that are potentially involved in common biological functions, typically determined by enrichment of annotation terms (4–7). Importantly, under this assumption enriched functions can be assigned to poorly annotated genes within the same co-expression module, an approach commonly referred to as ‘guilt by association’ (8). This approach allows a tentative prediction of function of genes that are novel or less studied, taking advantage of correlation patterns in the transcriptome expression configurations to infer a potential biologic function of uncharacterized genes, and also identifying new candidate interacting partners of known genes.

In addition to classical coding mRNAs, high-throughput sequencing (RNA-seq) discovers thousands of novel non-coding RNAs (ncRNAs), providing compelling evidence for the function of RNA beyond its role as messenger for protein-coding genes (9, 10). An important group of non-coding genes is represented by long non-coding RNAs (lncRNAs), transcripts without coding capacity that may interact with proteins, DNA, or other RNAs to perform structural and regulatory functions (11, 12). LncRNAs can be important regulators of the immune response, and recent publications have shown widespread changes in the expression of lncRNAs during the activation of the innate immune response and T cell development, differentiation, and activation (13). LncRNAs control important aspects of immunity such as production of inflammatory mediators, differentiation, cell migration. There is also emerging evidence suggesting that lncRNAs constitute a major subgroup of the interferon signaling target genes, and that the interferon response is subject to regulation by a large number of host- and pathogen-derived lncRNAs (14). However, the potential importance of lncRNAs in the immune response is only now emerging, and it is likely that there are many additional immune-related lncRNAs acting via multiple different mechanisms to be discovered. Notably, through co-expression network analysis, lncRNAs probable functions can be predicted and linked to biological pathways based on module sharing, i.e. by assigning functions according to the functional enrichment of coding transcripts in the same module (15).

We present results of a co-expression network analysis in the largest, to our knowledge, data set to date of RNA-seq WBC transcriptomes derived from 624 individuals from the ProgeNIA study (16, 17). We describe the modules of co-expressed genes, some of which reflect critical features of underlying cellular composition. Furthermore, by leveraging the availability of extensive immune-phenotyping of the ProgeNIA cohort of volunteers characterized by fluorescence-activated cell sorting (FACS) analyses (18), we are further able to associate modules to a wide range of circulating cell subtypes, as granulocytes, circulating dendritic cells, natural killer (NK), B cells, and T cells. In particular, T cells are subdivided according to their maturation and activation status, including subsets of regulatory T cells, allowing us to characterize T cells modules with unprecedented resolution.

We analyzed in total 15,807 gene-based transcripts, 1,798 of which are lncRNAs, providing a comprehensive view of transcriptome organization in human WBC and inferring possible functions for hundreds of these lncRNAs. In providing our network results, we also supply different tools that allow interrogation of the networks and extraction of important information on the complex inter-relationships identified in our analysis.

## Materials and methods

### The SardiNIA dataset

The 624 participants in this study are from four towns in the Lanusei Valley in the Ogliastra region of Sardinia, and were enrolled from the SardiNIA project (16), a longitudinal study of 6,921 general population individuals (57% females, 43% males), comprising related and unrelated individuals, ranging from 18 to 102 years. For 606 of these samples, extensive immune-phenotyping is available (18). Specifically, a wide range of circulating cell subtypes were characterized by fluorescence-activated cell sorting (FACS) analyses. The cells comprised the major leukocyte populations in peripheral blood, including monocytes, granulocytes, circulating dendritic cells, natural killer, B cells, and T cells, with a more detailed characterization of T cell subsets, subdivided according to their maturation and activation status, including subsets of regulatory T cells, resulting in a total of 95 cell types.

Gene-level expression values have been derived as described in (17). Briefly, RNA samples from white blood cells of the 624 individuals were enriched for PolyA(+) transcripts and processed with RNA-seq. Gene-level read counts (computed with GENCODE V14 annotation) were variance-stabilized with DESeq (19). Hidden factor estimation was performed with PEER (20), a factor analysis method that uses Bayesian approaches to infer hidden factors that explain a large proportion of expression variability, and 30 factors were used to compute the residuals. After excluding low expressed transcripts, we analyzed 15,807 gene-based transcripts (GENCODE V27), 1,798 of which are lncRNAs. We also analyzed 151 miRNA precursors, as the RNAseq data derived from a library of RNA fragments approximately 200 nucleotides in length, and this selection process does not capture mature miRNAs, which are shorter on average, around 25 nucleotides. We additionally fit a linear model with age and sex using SWAMP (21), as these factors were not the focus in this analysis, and derived residuals for the 621 individuals with available age information analyzed in the downstream network analysis.

### Co-expression networks

Network analysis was performed using the Weighted Gene Co-expression Network Analysis method (22), implemented in the R package WGCNA (23), the most widely used package for co-expression analysis (7), which performs best at defining the network structure. Co-expression networks use graph theory, where each node represents a gene and each edge represents the strength of the co-expression relationship between two genes, to identify co-expression modules using hierarchical clustering on a correlation network created from expression data.

In details, network construction proceeds along subsequent steps. In the first step the correlation matrix, constructed from pairwise Pearson correlations between all genes, is transformed into an adjacency matrix through a power beta (soft threshold power), so that only strong connections are considered. Two different types of networks can be constructed: signed or unsigned networks, based on how negative correlations between genes are converted into adjacencies. In a signed network, negative correlations are basically not considered. Conversely, in unsigned networks, adjacency is based on the absolute value of correlation, such that strong negative correlations are treated as strong connections. A signed method creates networks where biologically meaningful modules (such as those representing a specific biological process) are better separated (4). An unsigned network allows to cluster together positively and negatively correlated genes, which may be particularly interesting when ncRNAs are incorporated into the network, as, for instance, miRNAs are known to exert their function mainly through down-regulation of other genes (24), and this also holds true for some long intergenic non-coding RNAs (lincRNAs) (25). In this study we constructed both signed and unsigned networks using a power transformation for correlations into adjacencies in order to maximize scale free topology of 10 and 3, respectively. We used lower values for the power beta compared to the suggested values of 12 and 6 for signed and unsigned networks, respectively. This is due to the prior strong PEER correction used to remove hidden factors before computing residuals.

From the adjacency matrix, a topological overlap matrix (TOM), a pairwise measure of node inter-connectedness (similarity), is calculated. The TOM transformation replaces each adjacency by a normalized count of neighbors that are shared by any two genes. WGCNA identifies modules of co-expressed genes with high topological overlap, a pair-wise measure that describes the similarity of two genes co-expression relationships with all other genes in the network. Next, genes are hierarchically clustered using 1−TOM as the distance measure and modules (groups of co-expressed genes) are determined by using a dynamic tree-cutting algorithm, implemented in WGCNA (26). Hierarchical clustering iteratively divides each cluster into sub-clusters to create a tree with branches representing co-expression modules. The hybrid dynamic tree-cutting algorithm was used with a minimum module size of 30 and a *deepSplit* parameter of 2 to identify modules.

The global gene expression of a module can be summarized with a single representative expression profile, which is referred as the module eigengene (ME), computed from the first principal component of the expression values of all the genes assigned to each module. Modules whose eigengenes had a Pearson correlation greater than 0.8 were merged to reduce the number of highly correlated modules. Using MEs an important WGCNA metric for each gene can also be derived, the gene module membership (MM), calculated from the Pearson correlation between the specific gene expression profile and the ME of a given module.

We also constructed two additional signed networks, one considering only males (N=274) and one only females (N=347). Specifically, we derived residuals for 621 individuals fitting a linear model with age only, using SWAMP (21), and constructed two signed networks using a power transformation for correlations into adjacencies of 10, as in the signed network with all subjects.

### Modules annotation

Modules can be interpreted using several strategies. The most common method is functional enrichment analysis, used to identify and rank overrepresented functional categories for the genes within a module. Protein coding genes with absolute value of MM greater than 0.10 were entered in g:Profiler (27, 28), ordered by decreasing MM, for pathway enrichment based on gene ontology (GO) functional annotation, Reactome, and Kyoto Encyclopedia of Genes and Genomes (KEGG). In g:Profiler, gene lists may be interpreted as ordered lists where elements are in order of decreasing importance. The ordered query option is useful when the genes are placed in some biologically meaningful order. g:Profiler then performs incremental enrichment analysis with increasingly larger numbers of genes from the top of the list. This optimization procedure identifies specific functional terms that characterize the gene set as a whole. g:Profiler uses multiple testing correction algorithms for distinguishing significant results from random matches. We used the default g:SCS method in this study.

Modules were also annotated by measuring their enrichment with specific marker genes lists, using the WGCNA function *userListEnrichment* (29). This function measures list enrichment between inputted lists of genes (e.g. genes within the same module) and predefined collections of gene lists. Significant enrichment is measured using a hypergeometric test, and p-values are corrected for multiple testing using Bonferroni method.

More importantly, in this study, using the quantitative levels of the 95 immune cell types obtained by flow cytometry on fresh blood samples (FACS), available for 606 overlapping samples (18), we validate cell specific module annotations, and, notably, identify more specific cell sub-types, exploiting the significant correlations obtained from FACS counts with the MEs. The Student asymptotic p-values for correlations between the MEs and the FACS counts were calculated with the *corPvalueStudent* function in WGCNA and only significant terms after multiple testing corrections were considered.

### Identifying hub genes

Co-expression modules identified by WGCNA can include hundreds of genes, so it is important to identify highly inter-connected genes within a module (hub genes) that best explains its behavior (30). Intra-modular hubs are central to specific modules in the network, while inter-modular hubs are central to the entire network. Intra-modular hub genes are frequently more relevant to the functionality of networks than other nodes (31). Highly connected intra-modular hub genes tend to have high MM values to the respective module. The MM also describes the extent to which a gene conforms to the characteristic expression pattern of a module (32). The sign of the MM encodes whether the gene has a positive or a negative correlation with the ME. If the MM of a gene for a given module is close to 1 (or also −1 in an unsigned network), it means that the gene is highly correlated with the module, so it is highly connected to the other module genes. In an unsigned network, genes with negative MMs represent inversely expressed genes, i.e. genes that are negatively correlated to the (majority of the) module genes. In other words, genes that tend to be down-regulated when genes in the module are up-regulated. If MM is close to 0, then the gene is not reliably part of the module. We defined hubs genes those with MMs in absolute value in the top 90^th^ quantiles within a module. Genes with MMs in absolute value <0.10 are considered not clearly assigned to the specific module.

## Results

### Network construction and module annotation

**Figure 1:**
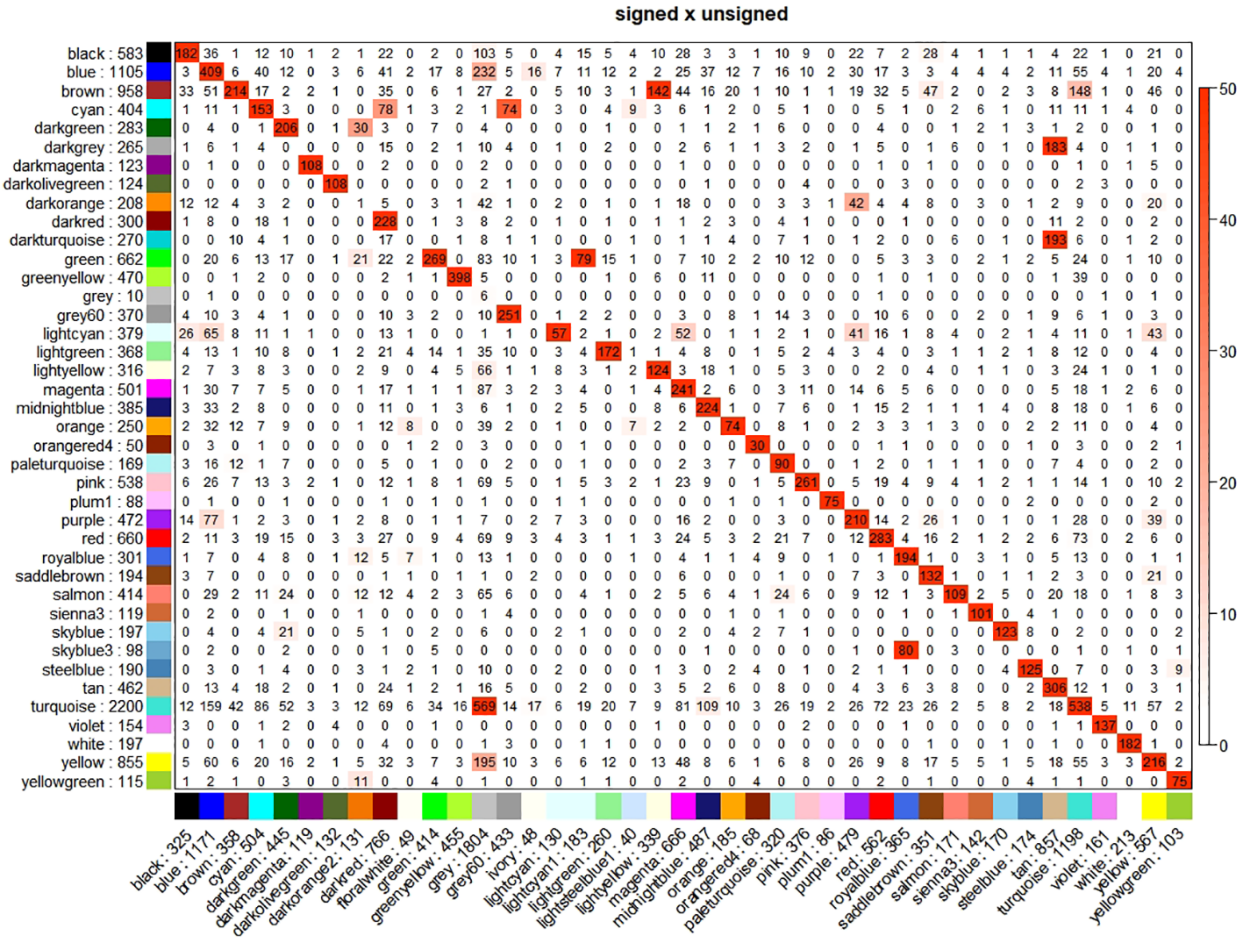
Cross-tabulations of modules of signed (column) vs. unsigned (row) networks. Coloring of the table encodes −log(p), with p being the Fisher’s exact test p-value for the overlap of the two modules. The stronger the red color, the more significant the overlap is.

We constructed both signed and unsigned networks using the WGCNA method. We identify 40 modules in the signed network and 41 modules in the unsigned network, highly overlapping (**Figure 1**). We describe here the main results for the unsigned network, highlighting the differences, when present, for the signed network.

**Figure 2:**
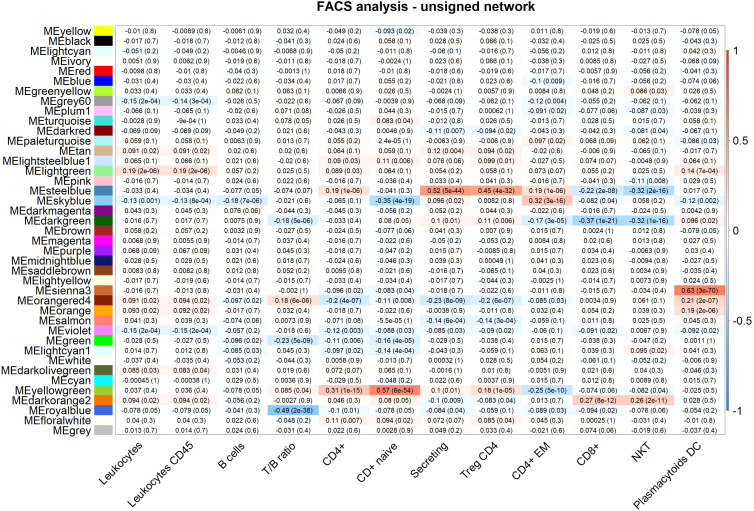
FACS analysis (selected traits) in the unsigned network. Module-trait heatmap displaying the correlation between the eigengene of a module (ME*, columns), identified in the unsigned network, and significant FACS counts (rows). Each cell contains the Pearson correlation coefficients which correspond to the cell color: red indicates a positive correlation, while blue indicates a negative correlation. Shading of colors encodes −log(p), with p being the significance of the correlation. The p-values are stated in the brackets.

**Figure 3:**
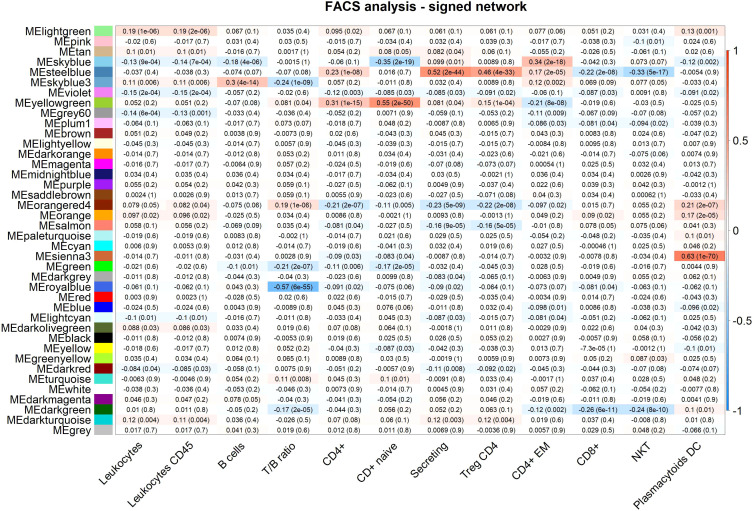
FACS analysis (selected traits) in the signed network. Module-trait heatmap displaying the correlation between the eigengene of a module (ME*, columns), identified in the signed network, and significant FACS counts (rows). Each cell contains the Pearson correlation coefficients which correspond to the cell color: red indicates a positive correlation, while blue indicates a negative correlation. Shading of colors encodes −log(p), with p being the significance of the correlation. The p-values are stated in the brackets.

We focus on 13 modules identified in the unsigned analysis (**Table 1**), closely related to 15 modules identified in the signed network (**Table 2**), that are closely associated with WBC or immune-related functions. Module annotation is carried out through enrichment analyses for immune-related functions, WBC marker genes, and also, in this study, by taking advantage of the availability of a broad immune characterization of specific cells subtypes measured on the same individuals. We use MEs to relate modules to FACS measurements by calculating Pearson correlations and significance between MEs and FACS counts (**Figures 2**, **3**, for the unsigned and the signed network, respectively). In the **Supplementary Data Sheet 1** we provide all genes included in this study with their module assignment, MM, and 1-quantile(MM), both for the signed and the unsigned networks. In the **Supplementary Data sheet 2** we provide all enrichments results obtained with the WGCNA predefined lists, and g:Profile analysis for each module, both for the signed and the unsigned networks.

**Table 1:** WBC and immune-related modules: top significant terms for the unsigned network.

Module	N unsigned	*N signed*	Description	G:Profiler ^(a)^		WGCNA^(b)^		FACS^(c)^		
				GO/KEGG terms	p-value	Pre-defined lists	p-value	Traits	r	p-value
**cyan**	504	*404*	**Interferon signaling**	Type I interferon signaling pathway	1.18E-29	IFN alpha/beta	2.71E-05	–	–	–
**darkred**	766	*300*		Response to interferon-gamma	2.10E-16	Antigen processing and presentation	2.74E-06	–	–	–
**lightgreen**	260	*368*	**Leukocytes**	Leukocytes activation	6.090E-25	–	–	Leukocytes	0.19	1.60E-06
darkturquoise*	857 (tan)	*270*	**Neutrophils**	Neutrophil mediated immunity	2.58E-18	Neutrophils	1.82E-08	–	–	–
**tan**		*462*								
darkgrey*		*265*								
**royalblue**	365	*301*	**B cells**	B cell activation	1.37E-07	B cell	3.21E-45	T/B ratio	-0.49	1.51E-38
**green**	414	*662*		B cell mediated immunity	3.73E-106	B cell	6.05E-17	T/B ratio	-0.23	4.72E-09
**yellowgreen**	103	*115*	**T cells**	–	–	CD4	1.50E-07	CD4+ naïve	0.57	6.34E-54
**steelblue**	174	*190*		Cytokine-cytokine receptor interaction	4.03E-09	CD4	3.89E-17	Secreting	0.52	4.97E-44
**skyblue**	170	*197*		Receptor activity	3.47E-07	*-*	*-*	CD4+ EM	0.32	3.05E-16
**darkorange2**	131	*-*		Cellular defense response	8.44E-04	NK cell	7.32E-07	NKT & CD8+	0.26	2.40E-11
**darkgreen**	445	*283*	**NK cells**	NKcell mediated cytotoxicity	3.09E-07	NK cell	1.22E-32	NK cell	0.44	2.01E-30
**grey60**	433	*370*		Cell communication	1.78E-05	–	–	NK HLA DR+	0.27	3.44E-12
**sienna3**	142	*119*	**Dendritic cells**	**-**	*-*	*-*	–	Plasmacytoids	0.63	2.73E-70

* Only present in the signed network.

(a) Enrichments with g:Profiler analysis, p-values are corrected for multiple testing;

(b) Enrichments with pre-made list sets included in WGCNA, p-values corrected for multiple testing. Link: https://www.rdocumentation.org/packages/WGCNA/versions/1.70-3/topics/userListEnrichment;

(c) Pearson correlation between the ME and the FACS counts. P-values are not corrected for multiple testing, but only significant terms after taking into account multiple testing are shown.

**Table 2:** WBC and immune-related modules: top significant terms for the signed network.

Module	N signed	*N unsigned*	Description	G:Profiler ^(a)^			WGCNA^(b)^		FACS^(c)^		
				GO/KEGG terms	p-value		Pre-defined lists	p-value	Traits	r	p-value
**cyan**	404	*504*	**Interferon signaling**	Type I interferon signaling pathway	2.78E-26		IFN alpha/beta	9.29E-06	–	–	
**darkred**	300	*766*		Interferon gamma signaling	1.26E-18		Antigen processing and presentation	1.06E-17	–	–	–
**lightgreen**	368	*260*	**Leukocytes**	Leukocytes activation	6.41E-25		–	–	Leukocytes	0.19	1.60E-06
**darkturquoise**	270	*857 (tan)*	**Neutrophils**	Immune response	1.31E-05		Neutrophils	0.0021	–	–	–
**tan**	462			–	–		–	–	–	–	–
**darkgrey**	265			Neutrophil activation	1.66E-18		–	–	–	–	–
**royalblue**	301	*365*	**B cells**	B cell activation	8.24E-07		B cell	3.68E-37	T/B ratio	-0.57	5.84E-55
**skyblue3**	98	*-*		–	–		B cell	1.16E-05	B cell	0.30	4.20E-14
**green**	662	*414*		B cell mediated immunity	1.26E-102		B cell	1.93E-10	T/B ratio	-0.21	2.25E-07
**yellowgreen**	115	*103*	**T cells**	–	–		CD4	1.54E-06	CD4+ naïve	0.55	1.80E-50
**steelblue**	190	*174*		Cytokine-cytokine receptor interaction	1.56E-08		CD4	2.12E-14	Secreting	0.52	1.61E-44
**skyblue**	197	*170*		Receptor activity	1.01E-07		CD4	0.0028	CD4+ EM	0.34	1.69E-18
**darkgreen**	283	*445*	**NK cells**	Natural killer cell mediated cytotoxicity	5.73E-10		NK cell	1.21E-60	NK cell	0.43	9.25E-29
**grey60**	370	*433*		Signaling	7.00E-07		–	–	NK HLA DR+	0.29	2.58E-13
**sienna3**	119	*142*	**Dendritic cells**	–	–		–	–	Plasmacytoids	0.63	1.42E-70

(a) Enrichments with g:Profiler analysis, p-values are corrected for multiple testing;

(b) Enrichments with pre-made list sets included in WGCNA, p-values corrected for multiple testing. Link: https://www.rdocumentation.org/packages/WGCNA/versions/1.70-3/topics/userListEnrichment;

(c) Pearson correlation between the ME and the FACS counts. P-values are not corrected for multiple testing, but only significant terms after taking into account multiple testing are shown.

By analyzing 15,807 gene-based transcripts (including 1,798 lncRNAs), we assigned to annotated module, 8,543 (54%) gene-based transcripts, of which 573 (32% of the total 1,798 lncRNAs included in the study) were lncRNAs (**Table 3**).

**Table 3:** Distribution of the 15,807 gene-based transcripts used in the analysis (GENCODE V27).

Gene type		N	Predicted function*	Hubs**
**protein coding**	protein coding	11864	7178	2450
	IG C gene	14	14	8
	IG D gene	25	7	–
	IG J gene	11	9	1
	IG V gene	100	98	52
	TR C gene	6	5	3
	TR D gene	2	1	1
	TR J gene	60	6	2
	TR V gene	84	16	1
**Total (protein coding)**		**12166**	**7334**	**2518**
**lncRNA**	3prime overlapping ncRNA	17	6	2
	antisense	42	15	6
	antisense RNA	825	224	44
	bidirectional promoter lncRNA	1	–	–
	lincRNA	604	237	50
	processed transcript	163	50	16
	sense intronic	89	18	1
	sense overlapping	57	23	4
**Total (lncRNA)**		**1798**	**573**	**123**
**other non-coding RNA**	misc RNA	102	13	3
	Mt rRNA	2	2	–
	Mt tRNA	6	2	–
	miRNA	151	35	7
	rRNA	19	3	1
	scaRNA	8	–	–
	snoRNA	132	15	4
	snRNA	104	7	3
	TEC (To be Experimentaly Confirmed)	4	–	–
**Total (ncRNA)**		**528**	**77**	**18**
**pseudogenes**	pseudogene	47	19	2
	IG C pseudogene	3	3	1
	IG J pseudogene	3	1	–
	IG V pseudogene	12	7	1
	TR J pseudogene	3	–	–
	TR V pseudogene	9	2	–
	polymorphic pseudogene	4	2	–
	processed pseudogene	845	412	69
	transcribed processed pseudogene	70	19	1
	transcribed unitary pseudogene	24	6	2
	transcribed unprocessed pseudogene	170	54	16
	translated processed pseudogene	1	1	–
	unitary pseudogene	2	1	1
	unprocessed pseudogene	122	32	3
**Total (pseudogenes)**		**1315**	**559**	**96**
**TOTAL**		**15807**	**8543**	**2755**

*Predicted function: genes with MM > 0.20 in modules with defined function; ** Hubs: genes with 1 − quantile(MM) < 0.10 in modules with defined function.

### Interferon signaling

Among the immune-related modules, two modules, the *cyan* and the *darkred*, are significantly enriched for interferon signaling pathways, both in the signed and in the unsigned networks. Specifically, the *cyan* module is significantly associated with “type I interferon signaling pathway” (p=1.18E-29), whereas the *darkred* module is significantly associated with “response to interferon-gamma” (p=2.10E-16). The MEs of the *cyan* and *darkred* modules are inversely correlated (r=-0.34, p-value=0.0290 in unsigned network, **Supplementary Table 2** in the **Supplementary Material**), and the cyan module is also inversely correlated with the *grey60* module (associated to NK HLA DR+ cells, see below), although not significantly, with similar results in the signed network (**Supplementary Table 2** in the **Supplementary Material**). In a signed network (where all genes in the module are positively correlated with the ME) a positive correlation between MEs means that most genes in the two modules follow the same expression patterns, whereas a negative correlation between the MEs implies that the genes in one module show opposite expression patterns of the genes in the other module. In an unsigned network, a positive correlation between MEs may also imply that genes have opposite behavior, since a module can contain genes negatively correlated with the ME.

In detail, enrichments for the *cyan* module in the unsigned network include “defense response to virus” (p=3.45E-37); “defense response to other organism” (p=5.38E-28); and “innate immune response” (p=1.39E-26). Kyoto Encyclopedia of Genes and Genomes (KEGG) pathway enrichments point to “Influenza A” (p=1.81E-11), “Herpes simplex infection” (p=2.28E-09), “Measles” (p=8.38E-09), and “Hepatitis C” (p=1.03E-06). Enrichment analysis using the WGCNA predefined list (through the WGCNA function *userListEnrichment* that allows enrichment analysis for different pre-made collections of marker genes) shows significance for the “IFN alpha/beta” pathway (p= 2.71E-05). The ME of the *cyan* module does not correlate significantly with any FACS count (none of the Pearson correlations between the ME of the *cyan* module and FACS measurements are significant). The *TNFSF13B* gene, encoding the cytokine BAFF (B-cell-activating factor) (33) is a very important gene within this module with MM is in the top 12% of all the genes in the *cyan* module, indicating that *TNFSF13B* is central in this interferon type I module and highly correlated to the other module genes.

The *darkred* unsigned module is significantly enriched for numerous GO terms, in particular “innate immune response” (p=2.21E-16); “response to cytokine” (p=7.20E-15); “cellular response to interferon-gamma” (p=1.72E-13); and “cytokine-mediated signaling pathway” (p=1.49E-12). The most significant KEGG pathway enrichment is for “Antigen processing and presentation” (p=8.80E-10). The *darkred* module is also enriched for genes associated with many infectious and autoimmune diseases, specifically, “Influenza A” (p=1.43E-06), “Tuberculosis” (p=2.98E-06), “Autoimmune thyroid disease” (p=1.03E-05); “Intestinal immune network for IgA production” (p=1.17E-05); “Asthma” (p=3.20E-05); “Type I diabetes mellitus” (p=4.07E-05); and “Inflammatory bowel disease (IBD)” (p=8.09E-05), as the module contains many HLA genes. The predefined WGCNA lists support enrichment for “Antigen processing and presentation” in the *darkred* module (p=2.74E-06). The *darkred* module does not correlate significantly with any FACS measurement.

### Leukocytes

Among the WBC-related modules, the *lightgreen* module is enriched for terms indicating neutrophil and more general leukocyte activation. GO enrichments for the *lightgreen* module are highly significant for “secretory granule” (p=2.14E-38), “secretory vesicle” (p=1.96E-36), and different specific neutrophils-associated terms such as “neutrophil activation involved in immune response” (p=1.04E-35), “neutrophil degranulation” (p=1.04E-35), “neutrophil activation” (p=2.00E-35), and “neutrophil mediated immunity” (p=2.64E-35). Different GO terms are also associated with leukocyte functions like “leukocyte degranulation” (p=2.71E-34), “leukocyte activation involved in immune response” (p=1.90E-32), and “leukocyte activation” (p=6.09E-25). No specific enrichments are observed with the predefined WGCNA lists but the *lightgreen* ME and FACS counts show significant correlations for “leukocytes” (r=0.19, p-value=1.60E-06), “granulocytes” (r=0.19, p-value=3.11E-06), and “monocytes” (r=0.19, p-value=1.08E-06).

### Neutrophils

In the unsigned network, enrichment for the *tan* module is related to “neutrophil mediated immunity” (p=2.58E-18), “myeloid leukocyte mediated immunity” (p=3.01E-18), “leukocyte degranulation” (p=4.47E-18), “myeloid cell activation involved in immune response” (p=7.52E-18), and “neutrophil activation involved in immune response” (p=1.25E-17) among the top GO enrichment terms. The predefined WGCNA lists identify significant enrichment for “Neutrophils” (p=1.82E-08). The tan module does not correlate significantly with any FACS measurement, as expected, since neutrophils are not included in the FACS panel.

The unsigned tan module is large and corresponds to three distinct modules in the signed network, the *darkturquoise* and *tan* modules overlapping with genes with positive MMs, and the *darkgrey* overlapping with genes with negative MMs in the unsigned *tan* module. Indeed, *darkgrey* is inversely correlated with the *darkturquoise* and *tan* modules, although the correlation is significant only with the *darkturquoise* module (r=-0.45, p=0.0040) (**Supplementary Table 2** in the **Supplementary Material**). In the signed network the *darkgrey* is significantly enriched for “neutrophil activation involved in immune response” (p=9.39E-19), “neutrophil degranulation” (p=9.39E-19), “neutrophil activation” (p=1.66E-18) among the top GO terms. The *darkturquoise* is enriched for “response to external stimulus” (p=3.05E-07), “secretion” (p=4.10E-06), and “immune response” (p=1.31E-05), whereas no significant enrichments are observed for the signed *tan* module. None of these modules correlate significantly with any FACS measurement.

### B cells

Two modules in the unsigned network, *royalblue* and *green*, are significantly enriched for B cell-related functions and for B cell marker genes. In particular, the *royalblue* module is associated with B cell activation and the *green* module with B cell mediated immunity. The *royalblue* module in the unsigned network corresponds to two modules in the signed network, the *royalblue* and the *skyblue3*. The MEs of the *royalblue* and of the *skyblue3* modules in the signed network show negative correlation (r=-0.34, p=0.0314). The unsigned *royalblue* module is enriched for GO terms like “B cell activation” (p=1.37E-07), “B cell receptor signaling pathway” (p=1.14E-05), and “B cell proliferation” (p=1.67E-05); and similarly for “B cell receptor signaling pathway” (p= 1.14E-05) among KEGG pathways. The predefined WGCNA lists indicates a highly significant enrichment in the *royalblue* module for genes associated with “B cell” (p=3.21E-45). Correlation between the *royalblue* module and FACS counts shows a significant negative correlation with T/B ratio (r=-0.49, p=1.51E-38). For the signed *skyblue3* module a significant negative correlation is observed for T/B ratio (r=-0.24, p=1.26E-09) and a positive one for B cell counts (r=0.30, p=4.20E-14).

The *green* module contains almost all immunoglobulin genes of the dataset, and is, as would be expected, highly significantly enriched for “antigen binding” (p=1.02E-182); “adaptive immune response” (p=3.14E-131); “humoral immune response mediated by circulating immunoglobulin” (p=1.03E-126); “complement activation” (p=2.69E-123); “protein activation cascade” (p=7.81E-120); “immunoglobulin mediated immune response” (p=1.20E-106); and “B cell mediated immunity” (p=3.73E-106). The predefined WGCNA lists also indicate a highly significant enrichment for marker genes associated with “B cell” (p= 6.05E-17). The *green* module is negatively correlated with T/B ratio in FACS (r=-0.23, p=4.72E-09).

### T cells

Four modules in the unsigned network are associated with T cells through enrichment analysis, corresponding to three modules in the signed network. For these modules FACS measurements are especially useful in suggesting T cell sub-classification according to their maturation and activation status. All FACS significant correlations with the T cells modules are shown in **Table 4** for the unsigned network, and in **Table 5** for the signed network.

**Table 4:** FACs results for T cells modules: Pearson correlation between the MEs of the T cells modules and FACs counts in the unsigned network.

FACs trait	Yellowgreen		Steelblue		Skyblue		Darkorange2	
	r	p-value*	r	p-value*	r	p-value*	r	p-value*
naive CD4+ AC	**0.57**	6.34E-54			-0.35	4.30E-19		
CD4+ AC	**0.31**	1.24E-15	**0.19**	1.40E-06				
CD4+ not Treg AC	**0.31**	1.85E-15	**0.18**	6.65E-06				
CD45RA+ CD25hi CD4+ not Treg AC	**0.29**	1.62E-13			-0.19	3.06E-06		
resting CD4+ Treg AC	**0.28**	1.52E-12			-0.18	4.33E-06		
naive CD8br AC	**0.19**	1.69E-06	-0.18	8.67E-06	-0.21	1.20E-07		
secreting CD4+ Treg AC			**0.52**	4.97E-44				
secreting & activated CD4+ Treg AC			**0.51**	4.12E-42				
CD25hi CD4+ AC			**0.51**	1.10E-41				
CD45RA- CD25hi CD4+ not Treg AC			**0.49**	2.01E-38	**0.27**	1.91E-11		
CM CD4+ AC			**0.45**	1.08E-32				
CD4+ Treg AC	**0.18**	9.54E-06	**0.45**	3.77E-32				
Resting & Secreting CD4+ Treg AC	**0.19**	2.56E-06	**0.45**	5.37E-32				
CD45RA- CD4+ AC			**0.39**	3.94E-24	**0.23**	7.07E-09		
CD45RA- CD4+ not Treg AC			**0.37**	1.17E-21	**0.24**	2.40E-09		
activated CD4+ Treg AC			**0.35**	1.43E-19				
CD25hi CD8br AC	-0.18	9.18E-06	**0.31**	1.34E-15				
CD39+ secreting CD4+ Treg AC			**0.26**	1.01E-10				
CD39+ CD4+Treg AC			**0.25**	4.10E-10				
CM CD8br AC			**0.25**	4.29E-10				
resting & activated CD4+ Treg AC	**0.24**	1.90E-09	**0.24**	2.47E-09				
CD39+ activated CD4+ Treg AC			**0.19**	1.44E-06				
CD4+ CD8br AC			**0.19**	2.02E-06				
CD39+ CD4+ AC			**0.18**	4.64E-06				
EM CD4+ AC	-0.25	5.45E-10	**0.19**	1.08E-06	**0.32**	3.05E-16		
CD28+ CD45RA- CD8dim AC					**0.22**	5.69E-08		
CD28- CD8br AC			-0.25	1.23E-10			**0.37**	2.90E-21
CD127- CD8br AC			-0.22	5.97E-08			**0.27**	6.73E-12
CD8+ AC			-0.22	2.02E-08			**0.27**	7.84E-12
NKT AC			-0.32	2.36E-16			**0.26**	2.40E-11
CD8br AC			-0.2	6.32E-07			**0.26**	2.70E-11
EM CD8br AC							**0.25**	2.47E-10
CD45RA- CD8br AC							**0.25**	4.57E-10
TD CD8br AC			-0.2	3.45E-07			**0.24**	7.01E-10
NKT CD8+ AC			-0.2	5.95E-07			**0.23**	4.86E-09
CD28- CD8dim AC							**0.23**	1.37E-08
CD28- CD4- CD8- AC							**0.22**	2.09E-08
TD CD4- CD8- AC			-0.23	4.91E-09			**0.21**	1.72E-07
CD45RA+ CD4- CD8- AC			-0.23	3.58E-09			**0.2**	3.15E-07
T lymphocyte AC							**0.19**	1.74E-06
TCR γδ AC			-0.21	9.43E-08			**0.19**	1.90E-06
NKT CD4- CD8- AC							**0.18**	3.80E-06

*p-values are not corrected, but only significant terms after taking into account multiple testing are shown. Bold values indicate positive correlations.

**Table 5:** FACs results for T cells modules: Pearson correlation between the MEs of the T cells modules and FACs counts in the signed network.

FACs trait	Yellowgreen		Steelblue		Skyblue	
	r	p-value*	r	p-value*	r	p-value*
naive CD4+ AC	**0.55**	1.80E-50			-0.35	1.55E-19
CD4+ AC	**0.31**	1.44E-15	**0.23**	1.27E-08		
CD4+ not Treg AC	**0.31**	2.04E-15	**0.21**	6.58E-08		
CD45RA+ CD25hi CD4+ not Treg AC	**0.27**	5.00E-12			-0.19	1.20E-06
resting CD4+ Treg AC	**0.27**	1.88E-11			-0.19	3.16E-06
naive CD8br AC	**0.2**	3.46E-07			-0.21	1.01E-07
T lymphocyte AC	**0.18**	9.40E-06				
secreting CD4+ Treg AC			**0.52**	1.61E-44		
secreting & activated CD4+ Treg AC			**0.51**	3.88E-42		
CD25hi CD4+ AC			**0.51**	7.53E-42		
CD45RA- CD25hi CD4+ not Treg AC			**0.48**	3.48E-37	**0.26**	9.21E-11
CM CD4+ AC			**0.47**	6.09E-36		
Resting & Secreting CD4+ Treg AC			**0.46**	1.42E-33		
CD4+ Treg AC			**0.46**	3.54E-33		
CD45RA- CD4+ AC			**0.39**	4.97E-24	**0.24**	1.39E-09
CD45RA- CD4+ AC			**0.38**	4.68E-23	**0.26**	2.61E-11
CD45RA- CD4+ not Treg AC			**0.37**	1.60E-21	**0.25**	4.15E-10
activated CD4+ Treg AC			**0.34**	9.83E-19		
CD4/CD8 ratio			**0.3**	2.83E-14		
CD25hi CD8br AC			**0.28**	5.26E-13		
CD39+ secreting CD4+ Treg AC			**0.26**	5.39E-11		
CD39+ CD4+Treg AC			**0.25**	2.46E-10		
resting & activated CD4+ Treg AC	**0.22**	3.57E-08	**0.25**	4.19E-10		
CM CD8br AC			**0.24**	2.14E-09		
CD39+ activated CD4+ Treg AC			**0.19**	1.01E-06		
CD39+ CD4+ AC			**0.19**	2.06E-06		
CD4+ CD8br AC			**0.19**	3.12E-06		
EM CD4+ AC	-0.21	7.71E-08			**0.34**	1.69E-18
CD28+ CD45RA- CD8br AC	-0.26	9.45E-11			**0.22**	4.43E-08
CD28+ CD45RA- CD8dim AC					**0.21**	1.47E-07

p-values are not corrected, but only significant terms after taking into account multiple testing are shown. Bold values indicate positive correlations.

The *yellowgreen* module, associated with CD4 T cells through the WGCNA predefined lists (p=1.50E-07), shows its most significant correlation with FACS naïve CD4+ count (r=0.57, p=6.34E-54). Thus the FACS sub-cell types correlating with this module indicate association with naïve CD4+ T cells.

The *steelblue* module is associated with CD4 T cells through the WGCNA predefined lists (p=3.89E-17), and is enriched for “Cytokine-cytokine receptor interaction” (p=4.03E-09) from the KEGG pathways, and for “C-C chemokine receptor activity” (p=5.37E-04); “cytokine-mediated signaling pathway” (p=6.99E-04); and “positive regulation of T cell differentiation” (p=1.66E-03) for GO terms in unsigned network. FACS analysis shows numerous T cell subtypes correlating with the module, with secreting count (secreting CD4+ Treg) as the top significant term (r=0.52, p=4.97E-44), indicating association of the *steelblue* module to regulatory CD4+ T cells. In agreement with FACS analysis results, this module has as a hub gene *FOXP3*, a master regulator in the development and function of regulatory T cells.

The *skyblue* module shows enrichment for “receptor activity” (p=3.47E-07) and “signaling receptor activity” (p=1.58E-06), and marginal enrichment for CD4 T cells from the WGCNA predefined lists (p=7.03E-03). FACS analysis shows a top significant correlation between *skyblue* and effector memory CD4+ T cells (r=0.32, p=3.05E-16).

Finally, in the unsigned network only, we observe an additional T cell module, the *darkorangered2* module, with suggestive enrichments pointing to “cellular defense response” (p=8.44E-04); “signaling receptor activity” (p=1.14E-03); “natural killer cell mediated immunity” (p=3.20E-03); and “antigen processing and presentation” (p=1.91E-03). FACS analysis allows us to correlate the module with natural killer T cells (r=0.26, p=2.40E-11) and cytotoxic CD8+ T cells (r=0.27, p=6.73E-12).

Pearson correlation analysis among the T cell modules show a significant negative correlation between the *yellowgreen* (naïve CD4+) and *skyblue* (EM CD4+) modules (r=-0.46, p=0.0027), and a significant positive correlation between the *steelblue* (regulatory CD4+) and the *skyblue* module (r=0.4, p=0.0090). The *darkorange2* module (cytotoxic CD8+ and natural killer T cells) shows a positive correlation with the *yellowgreen* module (r=0.35, p=0.0259), and negative correlation with the *skyblue* module (r=-0.36, p=0.0222).

### NK cells

We identify two modules associated with NK cells both in signed and unsigned networks, namely, the *darkgreen* and the *grey60* modules. The *darkgreen* module is significantly enriched for NK cell marker genes through the WGCNA predefined lists indicating NK cells (p=1.22E-32), and it is also associated with NK cell counts (r=0.44, p=2.01E-30) through FACS correlations, and CD3- lymphocyte counts (r=0.29, p=3.38E-13). Enrichments for the *darkgreen* module point to “receptor activity” (p=4.42E-09) and to “Natural killer cell mediated cytotoxicity” (p=3.09E-07). The *grey60* module shows enrichments for terms related to “cell communication” (p=1.78E-05), “signaling” (p=3.18E-05) and “vesicle” (p=6.90E-05). Correlation with FACS counts shows a significant correlation only for HLA DR+ NK counts (r=0.27, p=3.44E-12).

### Plasmacytoids dendritic cells

Although no significant enrichments are observed for the *sienna3* module, correlation with FACS shows highly significant correlation with plasmacytoid cDC cells (r=0.63, p=2.73E-70). Additional FACS counts correlated with the *sienna3* module are: CD86+ plasmacytoids (r=0.28, p=1.54E-12), CD62L plasmacytoids (r=0.23, p=5.04E-09), and dendritic cells (r=0.20, p=8.41E-07).

### LncRNA hub genes

Through the construction of a co-expression network including coding and non-coding genes, and enrichment analysis for coding genes in each module, we used the guilt–by–association approach to predict lncRNAs probable functions based on their co-expression with annotated protein-coding genes, as a foundation for further annotation and functional studies.

LncRNAs comprise any genes with a long non-coding gene biotype in the GENCODE v27 (i.e. ‘‘processed transcript’’, ‘‘sense intronic’’, ‘‘sense overlapping’’, ‘‘antisense’’, ‘‘lincRNA’’, ‘‘bidirectional promoter lncRNA’’, ‘‘3prime overlapping ncRNA’’). Among the 573 lncRNAs assigned to annotated modules, 123 lncRNAs are hub genes, defined as those with MMs in absolute value in the top 90^th^ quantiles within a module. As a validation of the power of the guilt–by–association approach, we searched scientific literature for the 55 hub lncRNA genes in the WBC and immune-related modules, finding confirming evidence from recent studies for the lncRNAs highlighted in **Table 6**.

**Table 6:** LncRNAs hub genes identified in WBC and immune-related modules.

Hub genes*	Gene type	Ref	Signed network			Unsigned network		
			Module	MM	1-q(MM)	Module	MM	1-q(MM)
***AL445490.1***	antisense RNA	(34–36)	cyan	0.62	0.06	cyan	0.68	0.05
*AP001610.1*	antisense RNA	–	cyan	0.55	0.1	cyan	0.58	0.07
***NRIR***	antisense RNA	(14, 37–45)	cyan	0.47	0.12	cyan	0.50	0.09
*AC244453.2*	antisense RNA	–	darkred	0.5	0.12	darkred	0.49	0.05
*LINC01887*	lincRNA	–	pink	0.12	0.54	darkred	-0.27	0.92
*RP11-229E13.2*	3prime overl. ncRNA	–	cyan	0.24	0.31	darkred	-0.28	0.92
*SCAMP1-AS1*	lincRNA	–	red	0.13	0.68	darkred	-0.30	0.94
***AC002546.1***	lincRNA	(46)	cyan	0.23	0.32	darkred	-0.32	0.95
*AC009948.1*	antisense RNA	–	cyan	0.31	0.23	darkred	-0.40	0.97
*AC104232.1*	lincRNA	–	lightgreen	0.79	0.04	lightgreen	0.81	0.06
*LINC01765*	lincRNA	–	tan	0.79	0	tan	0.66	0.00
*AC011444.2*	antisense RNA	–	tan	0.5	0.09	tan	0.49	0.02
*ADAMTSL4-AS1*	processed transcript	–	tan	0.46	0.1	tan	0.44	0.03
*AC020916.1*	lincRNA	–	tan	0.5	0.08	tan	0.39	0.06
*AC004069.1*	lincRNA	–	darkturquoise	0.23	0.69	tan	0.37	0.07
***LINC00963***	processed transcript	(47)	darkturquoise	0.31	0.49	tan	0.37	0.07
*AL356356.1*	antisense RNA	–	tan	0.38	0.18	tan	0.37	0.07
*AL353616.2*	lincRNA	–	tan	0.32	0.26	tan	0.35	0.09
*AC002511.2*	lincRNA	–	darkturquoise	0.24	0.67	tan	0.34	0.10
*AL354719.2*	antisense RNA	–	darkgrey	0.32	0.37	tan	-0.37	0.94
*LINC00671*	lincRNA	–	darkgrey	0.46	0.13	tan	-0.39	0.95
*AC005035.1*	lincRNA	–	darkgrey	0.36	0.29	tan	-0.39	0.95
*SLC12A5-AS1*	antisense RNA	–	darkgrey	0.69	0.01	tan	-0.59	1.00
***TCL6***	processed transcript	(48, 49)	royalblue	0.78	0.01	royalblue	0.81	0.01
***AL139020.1***	antisense RNA	(50)	royalblue	0.77	0.02	royalblue	0.79	0.02
***LINC00926***	lincRNA	(51)	royalblue	0.75	0.04	royalblue	0.74	0.04
***LINC02397***	lincRNA	(52)	royalblue	0.73	0.05	royalblue	0.70	0.05
***CTA-250D10.23***	lincRNA	(53)	royalblue	0.64	0.08	royalblue	0.61	0.08
*AL158850.1*	antisense RNA	–	skyblue3	0.61	0.1	royalblue	-0.29	0.91
*AC008074.3*	lincRNA	–	green	0.12	0.53	royalblue	-0.32	0.93
***AL928742.1***	lincRNA	(54)	skyblue3	0.8	0.02	royalblue	-0.34	0.95
***LINC01857***	lincRNA	(55)	skyblue3	0.68	0.08	royalblue	-0.22	0.81
*LINC02295*	lincRNA	–	yellowgreen	0.67	0.03	yellowgreen	0.67	0.04
*LEF1-AS1*	processed transcript	–	yellowgreen	0.66	0.04	yellowgreen	0.65	0.05
*WDR86-AS1*	processed transcript	–	skyblue	0.59	0.05	skyblue	0.61	0.05
***AL450992.2***	antisense RNA	(56)	skyblue	0.6	0.05	skyblue	0.60	0.06
*AL121748.1*	lincRNA	–	steelblue	0.63	0.02	steelblue	0.63	0.02
***MIR181A2HG***	antisense RNA	(57)	darkgreen	0.61	0.08	darkgreen	0.63	0.03
*AC018450.1*	processed transcript	–	darkgreen	0.53	0.13	darkgreen	0.55	0.07
*AC092535.1*	antisense RNA	–	darkgreen	0.57	0.11	darkgreen	0.51	0.09
*AC017100.1*	antisense RNA	–	darkgreen	0.51	0.14	darkgreen	0.49	0.09
***MIR155HG***	lincRNA	(58–61)	sienna3	0.1	0.92	darkgreen	-0.30	0.91
*MIAT*	lincRNA	–	turquoise	0.09	0.5	darkgreen	-0.32	0.92
***LINC02446***	lincRNA	(62)	salmon	0.2	0.35	darkgreen	-0.34	0.94
*LINC00943*	lincRNA	–	green	0.08	0.72	darkgreen	-0.37	0.95
*LINC00944*	lincRNA	–	green	0.11	0.59	darkgreen	-0.41	0.97
***LINC01871***	lincRNA	(63, 64)	turquoise	0.1	0.45	darkgreen	-0.43	0.97
*AC104809.2*	processed transcript	–	grey60	0.76	0.01	grey60	0.76	0.00
*AC100803.2*	sense overlapping	–	grey60	0.58	0.07	grey60	0.57	0.06
*AL590648.3*	lincRNA	–	grey60	0.55	0.09	grey60	0.56	0.07
*AC099552.2*	lincRNA	–	grey60	0.57	0.08	grey60	0.56	0.07
*AATBC*	antisense RNA	–	grey60	0.55	0.09	grey60	0.54	0.08
*LINC02345*	lincRNA	–	grey60	0.53	0.11	grey60	0.51	0.10
*AC097375.1*	lincRNA	–	sienna3	0.77	0.07	sienna3	0.78	0.06
*LINC00996*	lincRNA	–	sienna3	0.71	0.1	sienna3	0.71	0.08

* For lncRNAs genes in bold consistent functional evidence is found in published studies, cited in the Ref column.

By deriving for each gene its closest gene (the gene with highest adjacency, see Materials and Methods) within the same module in the signed networks, we observe multiple connections between the lncRNAs themselves. **Supplementary Table 3** in the **Supplementary Material** shows a summary by gene types (column) of their closest gene types (row) for all genes, and for important genes (considering a cutoff of 1-quantile(MM)<0.20) and adjacencies above the 3^rd^ quartile of the adjacencies for all genes (top adjacency>0.0338). Indeed, there are 398 lncRNAs that show the highest adjacency with other lncRNAs when considering all genes, and 15 lncRNAs showing strong adjacency with important lncRNAs.

### Additional network modules

Additional network modules, not associated to specific WBC cell-types or functionally related to the immune response, are associated to other cell-types, to general cell functions, or could not be clearly functionally characterized. In summary, we identified two modules associated to platelets, one module associated to reticulocytes, three modules associated to DNA metabolic processes, five modules associated to RNA metabolic processes (seven in the signed network), three modules associated to mitochondria (two in the signed network) and a module associated to the X-Y chromosomes. In the unsigned network we additionally identified a module associated to the endoplasmic reticulum, and two modules associated to cholesterol and lipid metabolic processes (**Supplementary Table 4** for the unsigned, and **Supplementary Table 5** for the signed network, in the **Supplementary Material**).

For instance, two modules, the *violet* and the *darkolivegreen*, are significantly enriched for platelets-related marker genes defined in the WGCNA predefined lists (p=9.34E-26 for the *violet*, and p= 9.31E-46 for the *darkolivegreen* module, respectively). Among the modules associated to DNA metabolic processes, the *plum1* module is significantly associated to “Cell Cycle” (p=2.90E-19), and “DNA Replication” (p=6.77E-16). The *lightcyan1* module is present only in the unsigned network, and it is positively correlated (r=0.37, p=0.0166) with the *green* module associated to “B cell mediated immunity”, and it is highly enriched for “Protein processing in endoplasmic reticulum” (p=5.33E-27) through the WGCNA predefined lists. Among the three modules associated to mitochondria in the unsigned network, the *lightyellow* module is highly enriched for numerous GO terms related to mitochondria, as “mitochondrial inner membrane” (p=1.99E-25), “mitochondrion” (p=1.50E-19), “catalytic complex” (p=7.17E-17). Interestingly, none of the genes in *lightyellow* module is located in the mitochondria DNA, whereas the *lightcyan* module in the unsigned network is constituted mainly by MT genes, pseudogenes, and non-coding genes.

### Sex-specific networks

We also constructed two signed networks for males and females, separately, in order to identify sex-specific hub genes/lncRNAs. We first looked at the modules overlap using the cross-tabulations of modules, and observed significant overlap across modules between the two sex-specific networks. **Supplementary Figure 1** in the **Supplementary Material** shows the module overlap for important genes (genes with 1-quantile(MM)<0.20) in the signed network with all individuals. We also looked for sex-specific hub genes/lncRNAs by examining the MM and 1-quantile(MM) differences for the modules that are closely associated with WBC or immune-related functions, and we did not observe any significant difference.

### Query tools

In providing our network results, we also supply different tools through the *Co-expression Network app* at cenb.irgb.cnr.it. These tools allow to interrogate the networks and to extract important information on the complex inter-relationships identified in our analysis.

In particular, *Tool 1* allows to identify the closest genes (i.e. genes with highest adjacency, a transformation of correlation, see Materials and Methods) to a specific input gene, within the module containing the input gene. It plots all the genes adjacencies (with the input gene) on the y-axis and their chromosomal position on the x-axis (**Supplementary Figure 2** in the **Supplementary Material**). A table is also created containing the list of genes in the module of the input gene, their adjacencies, MMs, and 1-quantile(MM)s.

With *Tool 2* it is possible to investigate whether a set of genes (e.g. genes associated through a GWAS to a specific trait or disease) are enriched in one or more modules. Significance is calculated through the Fisher’s exact test. This tool can be used to prioritize genes for further investigation or to validate the results.

With *Tool 3* it is possible to investigate a specific genomic region (e. g. a region identified through a GWAS) by plotting the network genes present in the region with their MMs on the y-axis and their chromosomal position on the x-axis. The legend indicates the functional modules the genes are associated to (**Supplementary Figure 3** in the **Supplementary Material**).

The assignment of genes to modules in a given network is unique in WGCNA: each gene is assigned to one module only (or to the *gray* module when assignment is indeterminate). However, a gene may be expressed in multiple cell-types or participate to multiple functional pathways. With *Tool 4*, it is possible to visualize the extent to which the gene conforms to the characteristic expression pattern of the network modules by plotting the input gene MMs in other modules (up to 10 top modules, considering only |MMs| > 0.10) (**Supplementary Figure 4** in the **Supplementary Material**).

## Discussion

Co-expression network analysis, through the observation of correlation of gene expression in transcriptomic data, has proven to provide reproducible results with biological relevance (65, 66). Genome-wide transcriptional network analysis is an unbiased, unsupervised approach, allowing hypothesis-free evaluation of how transcripts correlate with each other in a biological system of interest, such as the immune system, and allowing identification of modules of co-expressed genes that possess functional relevance.

We carried out the largest network analysis in human WBCs, using RNA-seq data derived from a sample of 624 individuals, and performed both signed and unsigned co-expression network analysis with WGCNA (23). We showed that the WBC transcriptome is organized into modules of co-expressed genes, including modules that reflect the underlying cellular composition. We were able to identify modules strongly related to specific immune cell-types (e.g. neutrophils, B and T cells, NK cells, and plasmacytoids dendritic cells), the interferon signaling pathways, and more general cellular functions, such as DNA and RNA metabolic processes and mitochondria functions. Indeed, genes that are most specifically and consistently expressed in the same cell-type appear highly correlated in transcriptome data, therefore gene co-expression clustering in heterogeneous tissues can be largely driven by cell composition effects (65, 67, 68). Nonetheless, cell-type-specific co-expression modules can be missed due to weak correlation in other cell-types (7).

Where the majority of network analyses assign biological meaning to modules by evaluating functional enrichment with specific marker genes lists and biological pathways, we have also, notably, exploited the availability of extensive immune-phenotyping of the cohort of volunteers characterized by FACS analyses to validate cell-type-specific module assignment obtained through enrichment analysis. Indeed, for a module consisting of cell-type-specific genes the module eigengene can be interpreted as a proxy for the relative number of relevant cells present in each sample. We are not aware of other studies that could associate modules to a wide range of circulating cell subtypes, as granulocytes, circulating dendritic cells, NK cells, B cells, and T cells (and sub-types) using this approach.

Moreover, within each module, we have identified and prioritized specific genes by identifying module hubs. These results lay a robust groundwork for subsequent experimental investigations aimed at delving deeper into the mechanisms governing gene regulation in human white blood cells.

Through RNA-seq technologies, it is now possible to interrogate the RNA expression levels of thousands of non-coding RNA transcripts (9, 10), like lncRNAs, for which there is emerging evidence suggesting that they are important regulators of the immune response (13). The identification and characterization of gene coexpression modules represent a powerful approach for annotating gene function of generally uncharacterized genes, like lncRNAs, and for generating hypotheses through the principle of guilt by association. Guilt by association implies that the expression levels of genes with the strongest evidence of membership for the same module are probably driven by the same underlying factors (69).

It is important to remember that not every gene in a module necessarily correlates with the functional annotation for which the module is enriched. The gene MM, that measure the extent to which the gene is inter-connected to the other genes in the module, can be used to relate the gene to the functional annotation of the module. Focusing on lncRNAs identified in the WBC and immune-related modules, and with high confidence for module assignment (we define hub genes those at the top 90^th^ quantiles of the MMs of all genes in the module), we found confirming evidence of their roles in the immune system in recent studies (**Table 6**). For instances, *AL445490.1* and *NRIR* are hub genes in a module associated with interferon signaling, and these lncRNAs are also found as important interferon target genes in recent studies (14, 35); The module associated with B cells contains as hub genes *TCL6* and *AL139020.1*, highly interconnected with the protein coding gene *TCL1A*, associated with pediatric B-cell acute lymphoblastic leukemia (48); In the same B cells module, but with opposite expression pattern, we highlight *AL928742.1*, a lincRNA closely co-expressed with *TBC1D27* and *TNFRSF13B* (*TACI*). *TACI* promotes T-cell independent antibody responses and plasma cell differentiation and counteracts *BAFF* driven B-cell activation (70).

In providing our network results, we also supply different tools that allow interrogation of the networks, in particular, a tool to identify the most connected genes to a specific gene of interest; a tool to investigate whether a set of genes (e.g., the genes identified in a GWAS) are enriched in one or more modules; a tool to plot all genes (and their modules) present in the network in a specific genomic region of interest (e. g., regions identified in a GWAS); and, finally, since genes may be expressed in multiple cell-types or participate in multiple functional pathways, a tool to provide a representation of the gene MMs across the network modules.

Although cellular heterogeneity is a major organizing principle in our gene co-expression networks study, some modules represent functional systems that span multiple cell-types, other modules capture variation in gene expression that is unrelated to cellular composition. For each identified module to map unambiguously to a specific cell-type, co-expression analysis should be performed in each cell-type separately (71). The organization of the human WBC transcriptome described here could not have been revealed by standard methods, such as differential expression analysis, as co-expression network analysis allows to identify relationships that are completely missed using more targeted approaches. It is important to highlight that co-expression networks are based only on correlations, therefore they indicate which genes are active at the same time across individuals, and thus likely in the same biological processes, but do not normally provide information about causality or distinguish between regulatory and regulated genes.

In conclusion, our network modules are rich sources of new hypotheses for thousands of genes expressed in WBCs, including lncRNAs, and provide valuable new resource for context-specific gene function annotation as a foundation for further mechanism studies.

## Data availability statement

Publicly available datasets were analyzed in this study. This data can be found here: https://ega-archive.org/datasets/EGAD00001003102.

## Ethics statement

The studies involving humans were approved by Ethical Committee of ASSL of Lanusei (2009/0016600) and Ethical Committee of ASSL of Sassari (2171/CE). The studies were conducted in accordance with the local legislation and institutional requirements. The participants provided their written informed consent to participate in this study.

## Author contributions

PF: Conceptualization, Formal analysis, Visualization, Writing – original draft, Writing – review & editing. MP: Conceptualization, Data curation, Visualization, Writing – original draft, Writing – review & editing, Investigation. FCr: Investigation, Writing – original draft, Writing – review & editing. MAD: Visualization, Software, Writing – review & editing. MM: Data curation, Investigation, Resources, Writing – review & editing. RC: Data curation, Investigation, Resources, Writing – review & editing. AA: Data curation, Investigation, Resources, Writing – review & editing. MS: Data curation, Investigation, Resources, Writing – review & editing. VO: Data curation, Investigation, Resources, Writing – review & editing. DS: Funding acquisition, Writing – review & editing. EF: Data curation, Investigation, Resources, Writing – review & editing. MD: Supervision, Writing – review & editing. FCu: Funding acquisition, Supervision, Writing – review & editing.

## Supplementary material

Supplementary Figure 1 Cross-tabulations of modules of males-only (column) vs. females-only (row) networks. Only genes with 1-quantile(MM)<0.20 in the signed network with all individuals are shown. Coloring of the table encodes −log(p), with p being the Fisher’s exact test p-value for the overlap of the two modules. The stronger the red color, the more significant the overlap is.

Supplementary Figure 2 Example of use of Tool 1. *Tool 1* allows to plot all genes in a module (the module of the input gene) with their adjacencies with the input gene. It plots the genes adjacencies on the y-axis and their chromosomal position on the x-axis, both for the signed and the unsigned networks. For example, we select as input gene *TNFSF13B*, which belongs to the *cyan* module (both in signed and unsigned networks), module associated with “Type I interferon signaling pathway”. A vertical blue line marks the position of *TNFSF13B*. The MM and 1-quantile of the MM of *TNFSF13B* is highlighted in the title. The color shading of the symbols is proportional with the MM of the gene in the module. We use the following symbols for the different gene types: red circle corresponds to mRNA; green triangle corresponds to ncRNA; blue triangle corresponds to miRNA; orange square corresponds to pseudogene.

Supplementary Figure 3 Example of use of Tool 3. With this tool it is possible to investigate specific genomic regions (e. g., regions identified in a GWAS). Selecting a genomic region in hg19 (human assembly GRCh37), for example 1:161036758-162036758, *Tool 3* allows to plot all the genes in the region present in the network, with their MMs on the y-axis and their chromosomal position on the x-axis, both for the signed and the unsigned networks. Colors of the plotted genes indicates the modules they belong to, and the legend indicates the modules functional annotations.

Supplementary Figure 4 Example of use of Tool 4. The assignment of genes to modules in a given network in WGCNA is univocal: each gene is assigned to one module only (or to the *gray* module when assignment is undefined). However, a gene may be expressed in multiple cell-types or participate in multiple functional pathways. With this tool it is possible to visualize the extent to which the gene conforms to the characteristic expression pattern of the network modules. *Tool 4* plots the input gene MMs in other modules (up to 10 top modules, considering only |MMs| > 0.10). We illustrate this tool for *TNFS13B* as input gene. Two bar plots are created by plotting the gene MMs in the signed and unsigned networks, respectively. Legends indicate the modules functional annotations.
