# Supplementary figures and images for: Transcriptome organization of white blood cells through gene co-expression network analysis in a large RNA-seq dataset
Source: Front Immunol. 2024 Apr 2;15:1350111. doi: 10.3389/fimmu.2024.1350111 (PMC11018966; doi:10.3389/fimmu.2024.1350111)

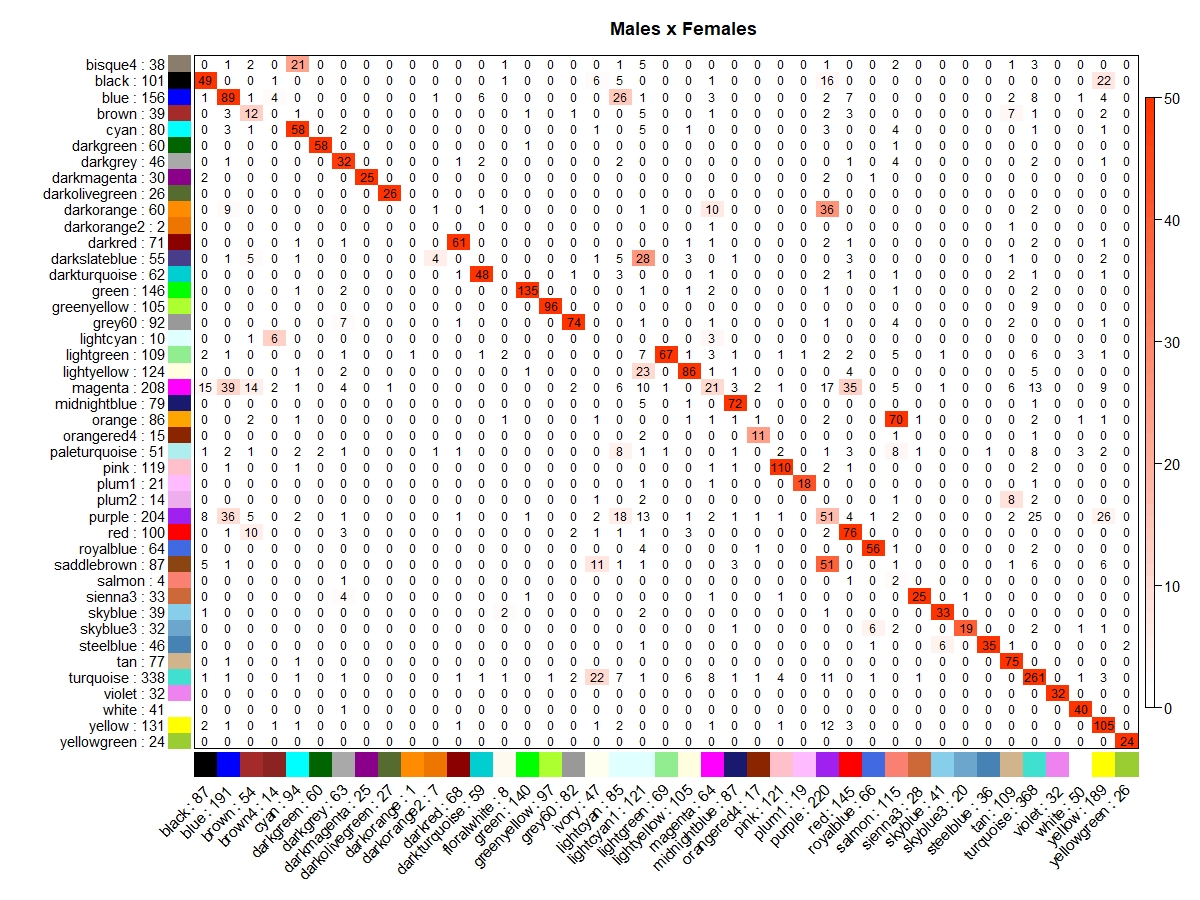

Supplement: Supplementary Figure 1 — Cross-tabulations of modules of males-only (column) vs. females-only (row) networks. Only genes with 1-quantile(MM)<0.20 in the signed network with all individuals are shown. Coloring of the table encodes −log(p), with p being the Fisher’s exact test p-value for the overlap of the two modules. The stronger the red color, the more significant the overlap is. [file Image_1.jpeg]

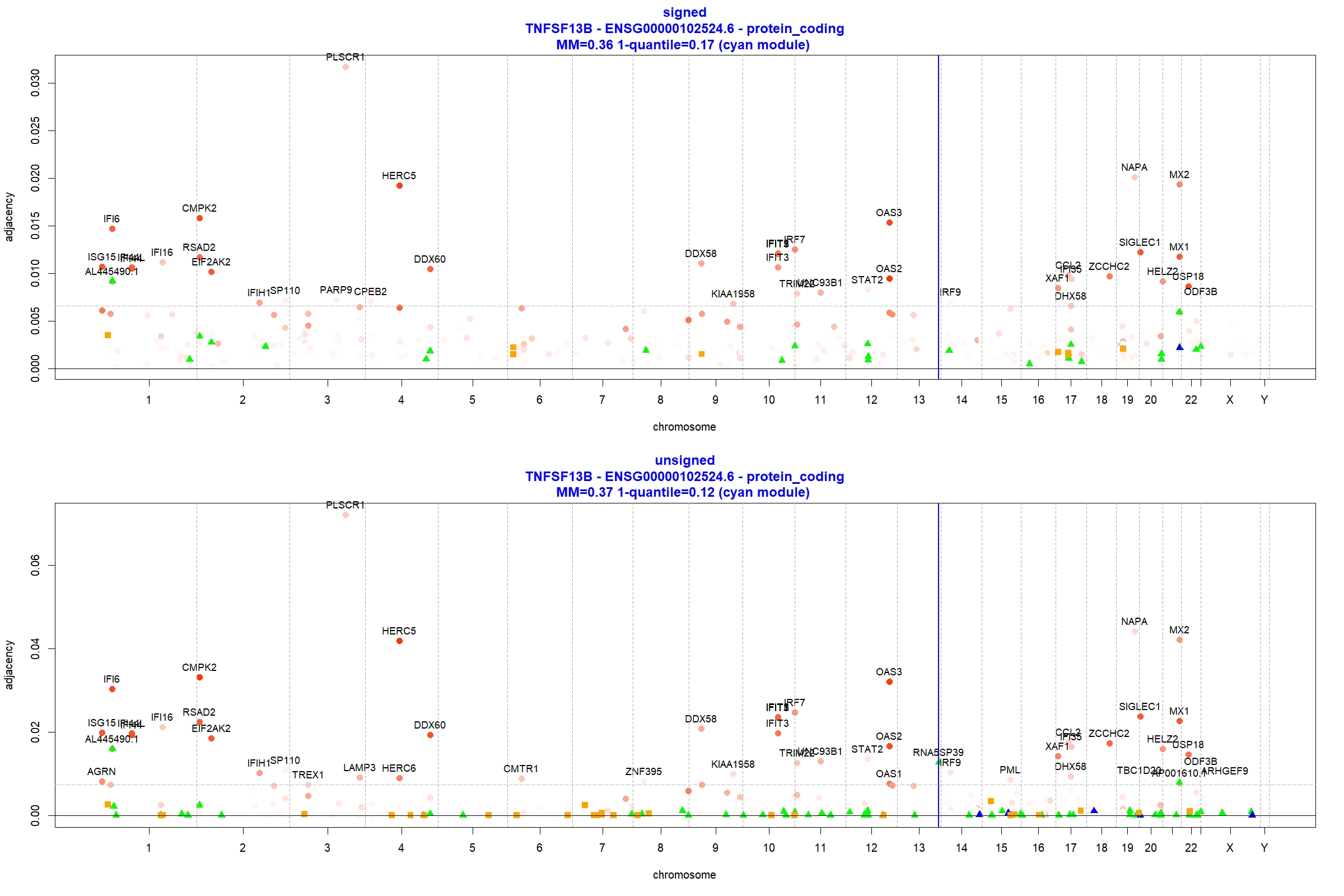

Supplement: Supplementary Figure 2 — Example of use of Tool 1. Tool 1 allows to plot all genes in a module (the module of the input gene) with their adjacencies with the input gene. It plots the genes adjacencies on the y-axis and their chromosomal position on the x-axis, both for the signed and the unsigned networks. For example, we select as input gene TNFSF13B, which belongs to the cyan module (both in signed and unsigned networks), module associated with “Type I interferon signaling pathway”. A vertical blue line marks the position of TNFSF13B. The MM and 1-quantile of the MM of TNFSF13B is highlighted in the title. The color shading of the symbols is proportional with the MM of the gene in the module. We use the following symbols for the different gene types: red circle corresponds to mRNA; green triangle corresponds to ncRNA; blue triangle corresponds to miRNA; orange square corresponds to pseudogene. [file Image_2.jpeg]

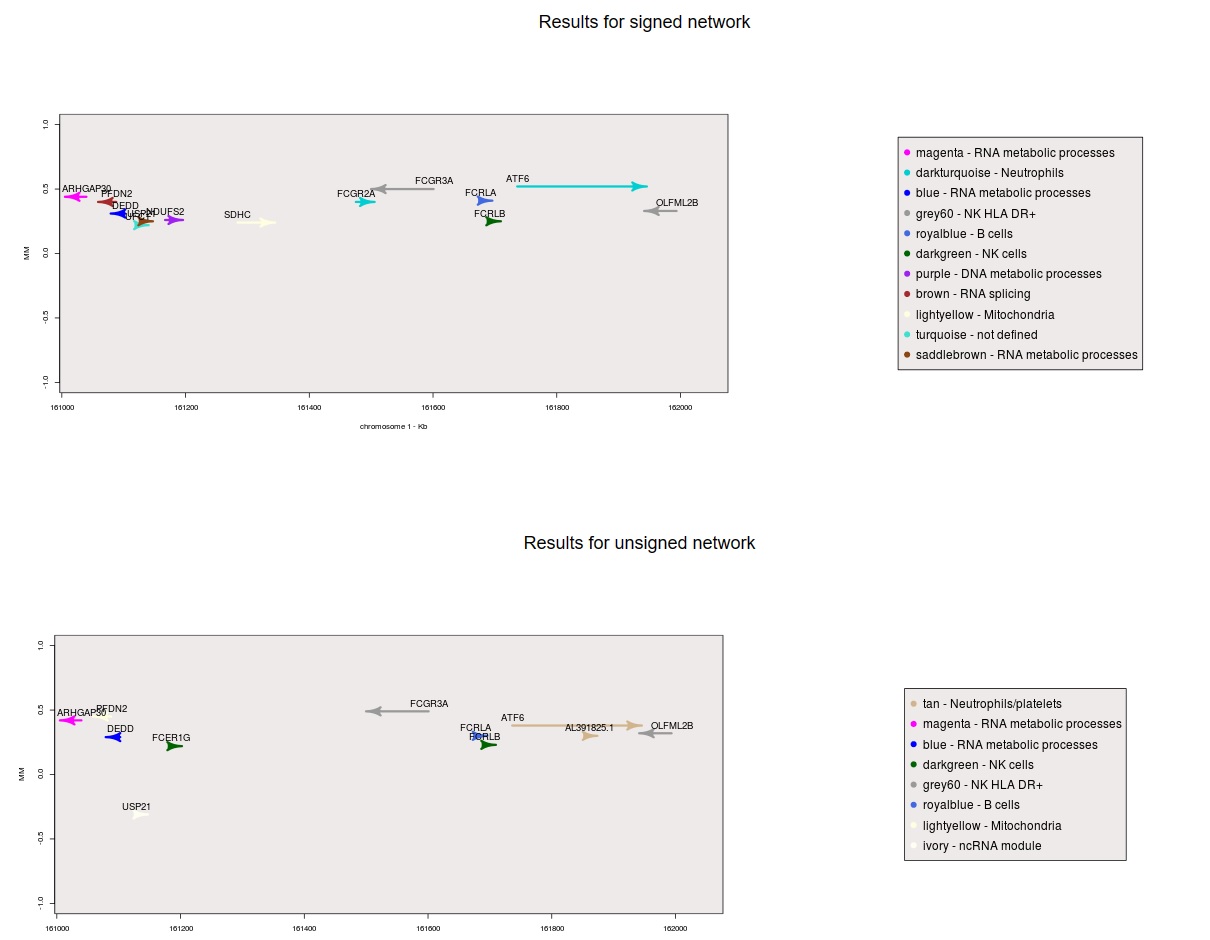

Supplement: Supplementary Figure 3 — Example of use of Tool 3. With this tool it is possible to investigate specific genomic regions (e. g., regions identified in a GWAS). Selecting a genomic region in hg19 (human assembly GRCh37), for example 1:161036758-162036758, Tool 3 allows to plot all the genes in the region present in the network, with their MMs on the y-axis and their chromosomal position on the x-axis, both for the signed and the unsigned networks. Colors of the plotted genes indicates the modules they belong to, and the legend indicates the modules functional annotations. [file Image_3.jpeg]

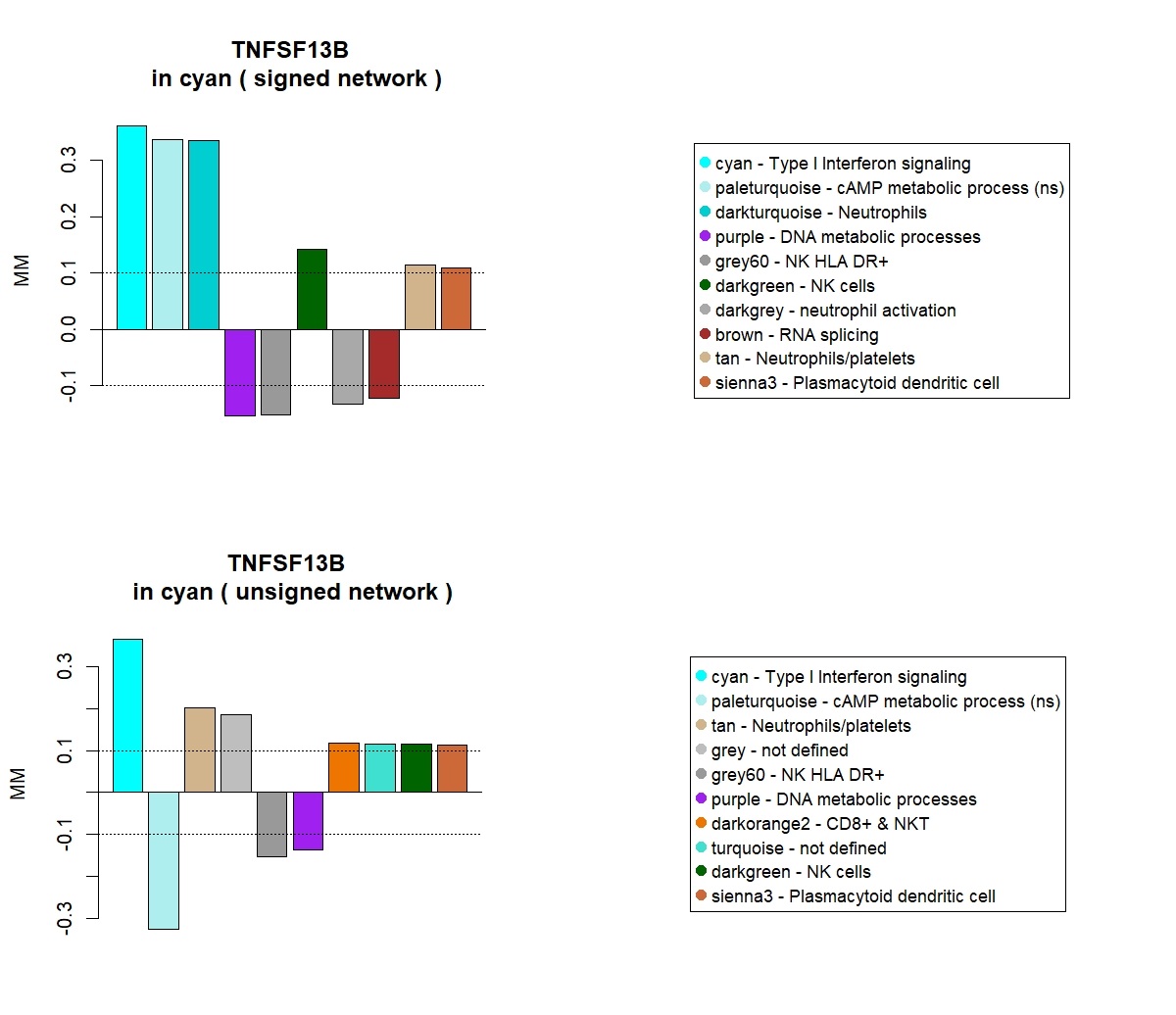

Supplement: Supplementary Figure 4 — Example of use of Tool 4. The assignment of genes to modules in a given network in WGCNA is univocal: each gene is assigned to one module only (or to the gray module when assignment is undefined). However, a gene may be expressed in multiple cell-types or participate in multiple functional pathways. With this tool it is possible to visualize the extent to which the gene conforms to the characteristic expression pattern of the network modules. Tool 4 plots the input gene MMs in other modules (up to 10 top modules, considering only |MMs| > 0.10). We illustrate this tool for TNFS13B as input gene. Two bar plots are created by plotting the gene MMs in the signed and unsigned networks, respectively. Legends indicate the modules functional annotations. [file Image_4.jpeg]
