# Supplementary material for: Transcriptome organization of white blood cells through gene co-expression network analysis in a large RNA-seq dataset
Source: Front Immunol. 2024 Apr 2;15:1350111. doi: 10.3389/fimmu.2024.1350111 (PMC11018966; doi:10.3389/fimmu.2024.1350111)
Supplement: Supplementary file 7 [file Table_1.docx]

**Supplementary Table 1 – Pearson correlations and nominal p-values between the modules eigengenes (MEs) of WBC/immune-related modules. Unsigned network.**

Right upper triangle shows Pearson correlations, left lower triangle shows nominal p-values

| **UNSIGNED NETWORK** | | **MElightgreen** | **MEtan** | **MEroyalblue** | **MEgreen** | **MEyellowgreen** | **MEsteelblue** | **MEskyblue** | **MEdarkorange2** | **MEdarkgreen** | **MEgrey60** | **MEsienna3** | **MEcyan** | **MEdarkred** |
| --- | --- | --- | --- | --- | --- | --- | --- | --- | --- | --- | --- | --- | --- | --- |
| **Module** | | **Leu** | **Neu** | **B cells** | | **T cells** | | | | **NK cells** | | **DC** | **Interferon signaling** | |
| **MElightgreen** | **Leukocytes** | - | 0.03 | -0.01 | -0.22 | 0.04 | -0.04 | -0.02 | -0.02 | -0.02 | -0.12 | 0 | 0.08 | -0.11 |
| **MEtan** | **Neutrophils** | ns | - | -0.13 | 0.02 | 0.05 | 0.13 | 0 | -0.02 | 0.09 | 0.01 | -0.09 | -0.02 | -0.15 |
| **MEroyalblue** | **B cells** | ns | ns | - | 0.14 | -0.06 | 0 | -0.06 | 0.22 | 0.06 | -0.01 | -0.07 | 0.04 | 0.03 |
| **MEgreen** |  | ns | ns | ns | - | -0.14 | 0.05 | 0.07 | 0.16 | -0.02 | -0.01 | 0.08 | 0.02 | -0.13 |
| **MEyellowgreen** | **T cells** | ns | ns | ns | ns | - | -0.04 | **-0.46** | **0.35** | 0.1 | 0.18 | -0.18 | -0.09 | -0.02 |
| **MEsteelblue** |  | ns | ns | ns | ns | ns | - | **0.4** | -0.27 | 0.2 | 0.03 | 0 | 0.05 | -0.1 |
| **MEskyblue** |  | ns | ns | ns | ns | 0.0027 | 0.0097 | - | **-0.36** | -0.02 | -0.05 | 0.01 | 0.06 | -0.02 |
| **MEdarkorange2** |  | ns | ns | ns | ns | 0.0259 | ns | 0.0222 | - | -0.23 | 0.01 | 0.07 | 0.09 | -0.11 |
| **MEdarkgreen** | **NK cells** | ns | ns | ns | ns | ns | ns | ns | ns | - | 0.01 | 0.03 | 0 | 0.07 |
| **MEgrey60** |  | ns | ns | ns | ns | ns | ns | ns | ns | ns | - | 0.04 | -0.24 | 0.12 |
| **MEsienna3** | **Plasmacytoids DC** | ns | ns | ns | ns | ns | ns | ns | ns | ns | ns | - | 0.13 | -0.06 |
| **MEcyan** | **Interferon signaling** | ns | ns | ns | ns | ns | ns | ns | ns | ns | ns | ns | - | **-0.34** |
| **MEdarkred** |  | ns | ns | ns | ns | ns | ns | ns | ns | ns | ns | ns | 0.029 | - |
