# Supplementary material for: Transcriptome organization of white blood cells through gene co-expression network analysis in a large RNA-seq dataset
Source: Front Immunol. 2024 Apr 2;15:1350111. doi: 10.3389/fimmu.2024.1350111 (PMC11018966; doi:10.3389/fimmu.2024.1350111)
Supplement: Supplementary file 8 [file Table_2.docx]

**Supplementary Table 2 – Pearson correlations and nominal p-values between the modules eigengenes (MEs) of WBC/immune-related modules. Signed network.**

Right upper triangle shows Pearson correlations, left lower triangle shows nominal p-values

| **SIGNED NETWORK** | | **MElightgreen** | **MEdarkturquoise** | **MEtan** | **MEdarkgrey** | **MEroyalblue** | **MEskyblue3** | **MEgreen** | **MEyellowgreen** | **MEsteelblue** | **MEskyblue** | **MEdarkgreen** | **MEgrey60** | **MEsienna3** | **MEcyan** | **MEdarkred** |
| --- | --- | --- | --- | --- | --- | --- | --- | --- | --- | --- | --- | --- | --- | --- | --- | --- |
| **Module** | | **Leu** | **Neutrophils** | | | **B cells** | | | **T cells** | | | **NK cells** | | **DCs** | **Interferon signaling** | |
| **MElightgreen** | **Leukocytes** | - | -0.04 | 0.08 | 0.01 | 0 | 0.11 | -0.27 | 0.06 | -0.03 | 0 | -0.01 | -0.14 | -0.03 | 0.13 | -0.13 |
| **MEdarkturquoise** | **Neutrophils** | ns | - | -0.02 | -0.29 | -0.04 | -0.01 | -0.07 | 0.07 | -0.01 | -0.12 | 0.2 | 0.05 | -0.03 | -0.1 | -0.09 |
| **MEtan** |  | ns | ns | - | **-0.45** | -0.1 | 0.01 | 0.03 | 0.1 | 0.19 | 0.02 | 0.03 | 0 | -0.02 | 0.04 | -0.1 |
| **MEdarkgrey** |  | ns | ns | 0.004 | - | 0.16 | 0.05 | 0 | 0.05 | -0.06 | 0 | -0.03 | 0.05 | 0.13 | 0.03 | 0.16 |
| **MEroyalblue** | **B cells** | ns | ns | ns | ns | - | **-0.34** | 0.1 | -0.01 | -0.01 | -0.05 | 0.12 | 0.04 | -0.08 | 0.07 | 0.06 |
| **MEskyblue3** |  | ns | ns | ns | ns | 0.0314 | - | -0.05 | 0.03 | -0.02 | 0.16 | -0.01 | 0 | 0.06 | 0.01 | 0.05 |
| **MEgreen** |  | ns | ns | ns | ns | ns | ns | - | -0.14 | 0 | 0.07 | 0 | -0.03 | 0.09 | 0.06 | -0.14 |
| **MEyellowgreen** | **T cells** | ns | ns | ns | ns | ns | ns | ns | - | 0 | **-0.48** | 0.1 | 0.18 | -0.18 | -0.04 | -0.05 |
| **MEsteelblue** |  | ns | ns | ns | ns | ns | ns | ns | ns | - | **0.36** | 0.13 | 0.07 | -0.03 | 0.03 | -0.07 |
| **MEskyblue** |  | ns | ns | ns | ns | ns | ns | ns | 0.0017 | 0.0226 | - | -0.07 | -0.04 | 0.02 | 0.1 | 0.01 |
| **MEdarkgreen** | **NK cells** | ns | ns | ns | ns | ns | ns | ns | ns | ns | ns | - | 0.04 | 0.06 | 0.06 | 0.07 |
| **MEgrey60** |  | ns | ns | ns | ns | ns | ns | ns | ns | ns | ns | ns | - | 0.03 | **-0.32** | 0.04 |
| **MEsienna3** | **DCs** | ns | ns | ns | ns | ns | ns | ns | ns | ns | ns | ns | ns | - | 0.14 | -0.06 |
| **MEcyan** | **Interferon signaling** | ns | ns | ns | ns | ns | ns | ns | ns | ns | ns | ns | 0.0408 | ns | - | **-0.44** |
| **MEdarkred** |  | ns | ns | ns | ns | ns | ns | ns | ns | ns | ns | ns | ns | ns | 0.0041 | - |
