# Supplementary material for: Transcriptome organization of white blood cells through gene co-expression network analysis in a large RNA-seq dataset
Source: Front Immunol. 2024 Apr 2;15:1350111. doi: 10.3389/fimmu.2024.1350111 (PMC11018966; doi:10.3389/fimmu.2024.1350111)
Supplement: Supplementary file 9 [file Table_3.docx]

## Supplementary Table 3 – Summary of the top adjacency genes by gene types

| **all genes** | | | | | |
| --- | --- | --- | --- | --- | --- |
| gene type | top adjacency gene type | | | | |
|  | lncRNA | mRNA | other ncRNA | pseudogene | *Total* |
| lncRNA | 398 | 1245 | 43 | 112 | *1798* |
| mRNA | 505 | 11394 | 34 | 233 | *12166* |
| ncRNA | 138 | 297 | 55 | 38 | *528* |
| pseudogene | 114 | 589 | 22 | 590 | *1315* |
| *Total* | *1155* | *13525* | *154* | *973* | *15807* |
| **important genes, strong adjacency** | | | | | |
| gene type | top adjacency gene type | | | | |
|  | lncRNA | mRNA | other ncRNA | pseudogene | *Total* |
| lncRNA | 15 | 65 | 1 | 5 | *86* |
| mRNA | 46 | 1798 | 2 | 26 | *1872* |
| ncRNA | 2 | 2 | 2 | 1 | *7* |
| pseudogene | 5 | 32 | 2 | 101 | *140* |
| *Total* | *68* | *1897* | *7* | *133* | *2105* |

Summary by gene types (column) of their closest (top adjacency) gene types (row). This summary is derived for all genes (above), and for a subgroup of important genes in the module (genes with 1-quantile(MM)<0.20), and selected strong adjacencies (only adjacencies above the 3^rd^ quantile of the adjacencies for all genes) (below).
