# Supplementary material for: Transcriptome organization of white blood cells through gene co-expression network analysis in a large RNA-seq dataset
Source: Front Immunol. 2024 Apr 2;15:1350111. doi: 10.3389/fimmu.2024.1350111 (PMC11018966; doi:10.3389/fimmu.2024.1350111)
Supplement: Supplementary file 10 [file Table_4.docx]

## Supplementary Table 4 – Other modules: top significant terms for the unsigned network

| **Module** | **N unsigned** | ***N signed*** | **Description** | **G:Profiler ^(a)^** | | **WGCNA^(b)^** | |
| --- | --- | --- | --- | --- | --- | --- | --- |
|  |  |  |  | **GO/KEGG terms** | **p-value** | **Pre-defined lists** | **p-value** |
| **darkolivegreen** | 132 | *124* | **Platelets** | Platelet degranulation | 6.73E-14 | Platelets | 9.31E-46 |
| **violet** | 161 | *154* |  | DNA packaging complex | 1.05E-27 | Platelets | 9.34E-26 |
| **pink** | 376 | *538* | **Reticulocytes** | Hemoglobin complex | 8.94E-15 | Reticulocytes | 1.39E-34 |
| **plum1** | 86 | *88* | **DNA metabolic processes** | Cell cycle process | 9.79E-52 | Cell Cycle | 2.90E-19 |
| **purple** | 479 | *472* |  | Chromosome organization | 5.75E-05 | - | - |
| **darkmagenta** | 119 | *123* |  | DNA binding | 7.40E-70 | Transcription pathway | 2.85E-62 |
| **yellow** | 567 | *855* | **RNA metabolic processes** | Nucleic acid metabolic process | 5.81E-13 | - | - |
| **saddlebrown** | 351 | *194* |  | RNA binding | 2.68E-24 | - | - |
| **blue** | 1171 | *1105* |  | RNA binding | 4.74E-12 | - | - |
| **red** | 562 | *660* |  | Regulation of RNA metabolic process | 3.26E-05 | - | - |
| **magenta** | 666 | *501* |  | RNA binding | 1.17E-08 | - | - |
| **lightcyan1** | 183 | *-* | **Endoplasmic reticulum** | Protein processing in endoplasmic reticulum | 1.34E-32 | Protein processing in endoplasmic reticulum | 5.33E-27 |
| **lightyellow** | 339 | *316* | **Mitochondria** | Oxidative phosphorylation | 3.91E-16 | Oxidative phosphorylation | 6.05E-17 |
| **lightcyan** | 130 | *379* |  | Respiratory chain | 4.53E-20 | - | - |
| **midnightblue** | 487 | *385* |  | Oxidative phosphorylation | 2.68E-06 | Mitocondria | 1.05E-06 |
| **lightsteelblue1** | 40 | *-* | **Metabolic process** | Cholesterol metabolic process | 1.54E-28 | Superpathway of cholesterol biosynthesis | 3.06E-19 |
| **floralwhite** | 49 | *-* |  | Lipid oxidation | 4.54E-10 | Fatty acid beta oxidation | 5.99E-03 |
| **orangered4** | 68 | *50* | **X-Y module** | Gonosomal inheritance | 3.18E-12 | - | - |

^(a)^ Enrichments with g:Profiler analysis, p-values are corrected for multiple testing;

^(b)^ Enrichments with pre-made list sets included in WGCNA, p-values corrected for multiple testing. Link: <https://www.rdocumentation.org/packages/WGCNA/versions/1.70-3/topics/userListEnrichment>
