# Supplementary material for: Transcriptome organization of white blood cells through gene co-expression network analysis in a large RNA-seq dataset
Source: Front Immunol. 2024 Apr 2;15:1350111. doi: 10.3389/fimmu.2024.1350111 (PMC11018966; doi:10.3389/fimmu.2024.1350111)
Supplement: Supplementary file 11 [file Table_5.docx]

## Supplementary Table 5 – Other modules: top significant terms for the signed network

| **Module** | **N signed** | ***N unsigned*** | **Description** | **g:Profiler ^(a)^** | | **WGCNA^(b)^** | |
| --- | --- | --- | --- | --- | --- | --- | --- |
|  |  |  |  | **GO/KEGG terms** | **p-value** | **Pre-defined lists** | **p-value** |
| **darkolivegreen** | 124 | *132* | **Platelets** | Platelet degranulation | 3.12E-13 | Platelets | 5.42E-43 |
| **violet** | 154 | *161* |  | DNA packaging complex | 4.37E-24 | Platelets | 9.34E-26 |
| **pink** | 538 | *376* | **Reticulocytes** | Hemoglobin complex | 7.50E-15 | Reticulocytes | 4.51E-31 |
| **plum1** | 88 | *86* | **DNA metabolic processes** | Cell cycle process | 9.67E-49 | Cell Cycle | 7.18E-18 |
| **purple** | 472 | *479* |  | DNA repair | 7.57E-06 | - | - |
| **darkmagenta** | 123 | *119* |  | DNA binding | 2.08E-79 | Transcription pathway | 5.26E-66 |
| **brown** | 958 | *358* | **RNA metabolic processes** | RNA splicing | 9.58E-20 | Spliceosome | 7.58E-12 |
| **darkorange** | 208 | *-* |  | Chromatin organization | 1.17E-09 | - | - |
| **yellow** | 855 | *567* |  | Nucleic acid metabolic process | 2.00E-07 | - | - |
| **saddlebrown** | 194 | *351* |  | RNA binding | 2.75E-15 | - | - |
| **blue** | 1105 | *1171* |  | Nucleic acid binding | 3.64E-17 | - | - |
| **red** | 660 | *562* |  | Transcription from RNA polymerase II promoter | 1.24E-04 | - | - |
| **magenta** | 501 | *666* |  | RNA binding | 3.71E-05 | - | - |
| **lightyellow** | 316 | *339* | **Mitochondria** | Oxidative phosphorylation | 5.90E-28 | Electron transport chain | 1.66E-23 |
| **lightcyan** | 379 | *130* |  | tRNA processing in the mitochondrion | 8.25E-17 | - | - |
| **orangered4** | 50 | *68* | **Gonosomal inheritance** | Gonosomal inheritance | 1.76E-08 | - | - |

^(a)^ Enrichments with gProfiler analysis, p-values are corrected for multiple testing;

^(b)^ Enrichments with pre-made list sets included in WGCNA, p-values corrected for multiple testing

Link: <https://www.rdocumentation.org/packages/WGCNA/versions/1.70-3/topics/userListEnrichment>
